# Untargeted metabolomics of cervicovaginal fluid in patients with ovarian cancer

**DOI:** 10.1017/cts.2026.10766

**Published:** 2026-06-22

**Authors:** Oyomoare L. Osazuwa-Peters, April Deveaux, Brandon Eudy, Temitope Keku, Andrew Berchuck, Ashwini Joshi, Bin Huang, Thomas Tucker, Kevin Ward, Maria Pisu, Margaret Gates Kuliszewski, Rebecca A. Previs, Tomi Akinyemiju

**Affiliations:** 1 Population Health Sciences, Duke University School of Medicine, Durham, NC, USA; 2 Metabolon Inc, Morrisville, NC, USA; 3 The University of North Carolina, Chapel Hill, NC, USA; 4 Gynecologic Oncology, Duke University School of Medicine, Durham, NC, USA; 5 Kentucky Cancer Registry, Lexington, KY, USA; 6 University of Kentucky, Lexington, KY, USA; 7 Georgia Cancer Registry, Atlanta, GA, USA; 8 The University of Alabama, Birmingham, AL, USA; 9 New York State Department of Health, Albany, NY, USA; 10 Labcorp, Durham, NC, USA

**Keywords:** Cervicovaginal fluid, ovarian cancer, metabolomics, population health, self-collection

## Abstract

Cervicovaginal fluid (CVF) represents a promising biospecimen for ovarian cancer biomarker discovery, but metabolomics typically requires specialized collection methods. We assessed the feasibility of applying untargeted metabolomics to self-collected CVF from 10 ovarian cancer patients in the ORCHiD (Ovarian Cancer Epidemiology, Healthcare Access and Disparities) study using ultrahigh performance liquid chromatography-tandem mass spectroscopy. We detected 1107 compounds mapping to 1002 unique metabolite identifiers across 9 super chemical classes, with detection rates of 62–99%. One-third of detected metabolites overlapped with published studies, while two-thirds were novel. Sample-level detection profiles were broadly consistent. Untargeted metabolomics on self-collected CVF is technically feasible and enables metabolite discovery for population-based cancer research.

## Introduction

Metabolomics, the high-throughput analysis of small molecular weight compounds in biological specimens, is increasingly applied to characterize biomarkers for cancer detection, prognosis, and treatment [1]. Metabolomics can be classified as targeted or untargeted, depending on whether analysis is done agnostically or focuses on predefined compound classes [1]. A major advantage of untargeted metabolomics lies in its potential to detect novel biomarkers of cellular processes closely connected to the phenotype of interest [2]. Consequently, untargeted metabolomics is increasingly applied in cancer research; between June 2010 and January 2026, there were 1,760 published articles on PubMed using the keywords untargeted metabolomics and cancer.

While blood, saliva, and urine have been subjected to untargeted metabolomics profiling, there is growing interest in less commonly studied complex body fluids, particularly those proximally located to the tumor microenvironment [2]. This is especially relevant for gynecological cancers such as ovarian cancer, where cervicovaginal fluid (CVF) represents a promising biospecimen. CVF is produced as a protein-rich fluid in the fallopian tube, actively transports oocytes from the ovary to the endometrium, and drains down the endometrial cavity through the cervix into the vagina [3]. For ovarian cancer, characterizing the metabolic profile of CVF could provide an opportunity to detect changes in the fallopian tube and ovarian tumor microenvironment [3]. However, clinic-based biospecimen collection presents substantial challenges: it is logistically complex, requires additional clinical workflow integration, may introduce selection bias by focusing primarily on patients already receiving treatment, and has historically lacked diverse population representation [4,5]. These barriers limit the feasibility of collecting fresh CVF samples specifically for metabolomics studies, particularly in large, population-based investigations. Self-collected samples obtained within epidemiologic studies offer a promising alternative, potentially yielding more representative biospecimens across diverse patient populations while bypassing the logistical constraints of clinic-based collection [5,6]. Yet, questions remain about whether metabolomics can be validly applied to samples collected using protocols not initially optimized for metabolic profiling.

Here, we describe the process and outcomes of untargeted metabolomics nested within a population-based ovarian cancer study with recruitment from state cancer registries across 7 US states (New York, Kentucky, California, North Carolina, Georgia, Maryland, and Texas). Our goal was to assess whether applying untargeted metabolomics on CVF samples from the ORCHiD (Ovarian Cancer Epidemiology, Healthcare Access and Disparities) study is feasible and can yield biological insights.

## Methods

### Study Participants

We randomly selected 10 individuals from the ORCHiD study who agreed and provided CVF between March 2021 and September 2022 [7]. Participants were all females at birth, 30 years or older, recruited 6-9 months after primary treatment, and provided written informed consent. The study was approved by the Duke University Institutional Review Board (Pro00101872).

### Biospecimen collection

Standardized written instructions were provided with each kit to minimize variability in collection technique. Participants self-collected CVF using the DNA Genotek OMR-130 kit, inserting vaginal swabs into tubes with stabilizing solution [7]. Samples were returned by mail, then cataloged, processed, and stored at Duke Molecular Physiology Institute. CVF samples were processed within 30 days by adding 5 µL Proteinase K (80 mg/mL), vortexing, incubating, aliquoting, and storing at −80°C [8].

### Metabolomics analysis

We aliquoted 100 μL of each sample into 2D barcoded tubes and shipped with dry ice to Metabolon (Morrisville, NC) for untargeted metabolomics. Range finding on 10 pilot samples and one blank collection kit was conducted to determine technical and scientific feasibility. Samples were accessioned into the Metabolon Laboratory Information Management System, assigned unique identities, and maintained at −80°C until processing. Processing involved automated preparation using the MicroLab STAR system (Hamilton Company), adding recovery standards, precipitating proteins with methanol, centrifugation, and removing organic solvent with a Zymark TurboVap (Hopkinton, MA). Extracts were divided into fractions for analysis by two separate reverse phase methods, with others reserved for backup. Extracts were stored overnight under nitrogen.

Two quality control sample types were analyzed concurrently with all study samples throughout the platform run: (1) MTRX: an extensively characterized human plasma pool maintained by Metabolon, used to verify that all aspects of the analytical process are operating within specifications; and (2) PRCS: ultra-pure water process blanks, used to assess background compound signals from the extraction process. Instrument variability, assessed by the median relative standard deviation (RSD) of internal standards added prior to injection, was 3%. Overall process variability, assessed by the median RSD of endogenous biochemicals in the MTRX replicates, was 8%. Both values met Metabolon’s acceptance criteria. Individual study samples were not run in technical replicate, consistent with the range-finding design of this pilot study.

Ultrahigh performance liquid chromatography-tandem mass spectroscopy (UPLC-MS/MS) was conducted using a Thermo Scientific Q-Exactive high resolution mass spectrometer with heated electrospray ionization source and Orbitrap mass analyzer operated at 35,000 mass resolution [9]. Raw data were extracted, peak-identified, and quality control processed using Metabolon software. Compounds were identified by comparison to library entries using three criteria: retention index within a narrow window, accurate mass match within ±10 ppm, and MS/MS forward and reverse scores between study data and authentic standards. Metabolon’s library contains 5400 purified or in-house synthesized standards. Peaks were quantified using area under the curve. An additional 7000 mass spectral entries for structurally unnamed biochemicals identified by recurrent chromatographic and mass spectral characteristics were included. Data normalization corrected instrument inter-day tuning variation. Data underwent quality control and curation to ensure accurate identification and remove system artifacts, mis-assignments, mis-integration, and background noise. Metabolite presence/absence data were filtered to exclude compounds detected in the blank biospecimen collection kit.

### Metabolite mapping

Metabolite presence/absence data were summarized by super classes and sub pathways. To evaluate cross-study reproducibility, metabolite lists were extracted from two published CVF metabolomics studies: Srinivasan et al. 2015 (*n*_Srinivasan_∼ = 280) and Ilhan et al. 2019 (*n*_Ilhan_∼ = 296) [10,11]. All metabolites were mapped to standardized RefMet identifiers [12,13] for cross-study comparison (*n*_Srinivasan_ = 273; *n*_Ilhan_ = 222; *n*_Pilot_ = 1002). RefMet standardization was performed using the current database (accessed April 2026); the updated mapping yielded 1,002 unique identifiers compared to 996 in the original analysis, reflecting minor updates to the RefMet database. Overlap was visualized using Venn diagrams [14].

### Statistical analysis

Sample-level detection profiles were characterized using binary (presence/absence) matrices. Hierarchical clustering of samples was performed using Jaccard distance with Ward D2 linkage, an approach appropriate for binary data as it considers only shared presences and does not penalize shared absences, the methodologically correct choice for swab-based data where absence may reflect detection limits rather than true biological absence. Sørensen distance was used as a sensitivity check, with the topological identity of the two dendrograms assessed with cophenetic correlation (≥0.75 indicates similarity). Multiple correspondence analysis (MCA) was performed on binary metabolite detection profiles using the MCA function in the *FactoMineR* package [15], including the blank collection kit as a quality control reference to confirm that retained compounds represent donor-derived biological signal rather than kit background artifacts. Hierarchical clustering dendrograms were visualized using the *ggdendro* package [16] and heatmaps were rendered using *pheatmap* [17] and converted to *ggplot* [18] objects using *ggplotify* [19] for composite figure assembly with *patchwork* [20]. Analyses were completed in R statistical computing software version 4.5.0 [21].

## Results

Mean age at diagnosis was 46.40 (SD: 14.67) years. All participants identified as non-Hispanic; 70% identified as White and 30% as Black. Insurance status, used as a proxy for socioeconomic status, was 70% private, 20% Medicare, and 10% unknown. Cancer characteristics included stage at presentation (50% localized, 40% distant, 10% regional), tumor type (50% Type I, 40% Type II, 10% other), and pathological grade (30% well differentiated, 20% poorly differentiated, 10% high grade, 40% unknown); all tumors were malignant. All participants had surgery and 80% received chemotherapy. Untargeted metabolomics analysis successfully detected a total of 1,107 distinct chemical compounds across all 10 CVF samples. These compounds mapped to 1,002 unique metabolite identifiers in the RefMet database [12,13] and spanned 9 super chemical classes.

**Figure 1.**
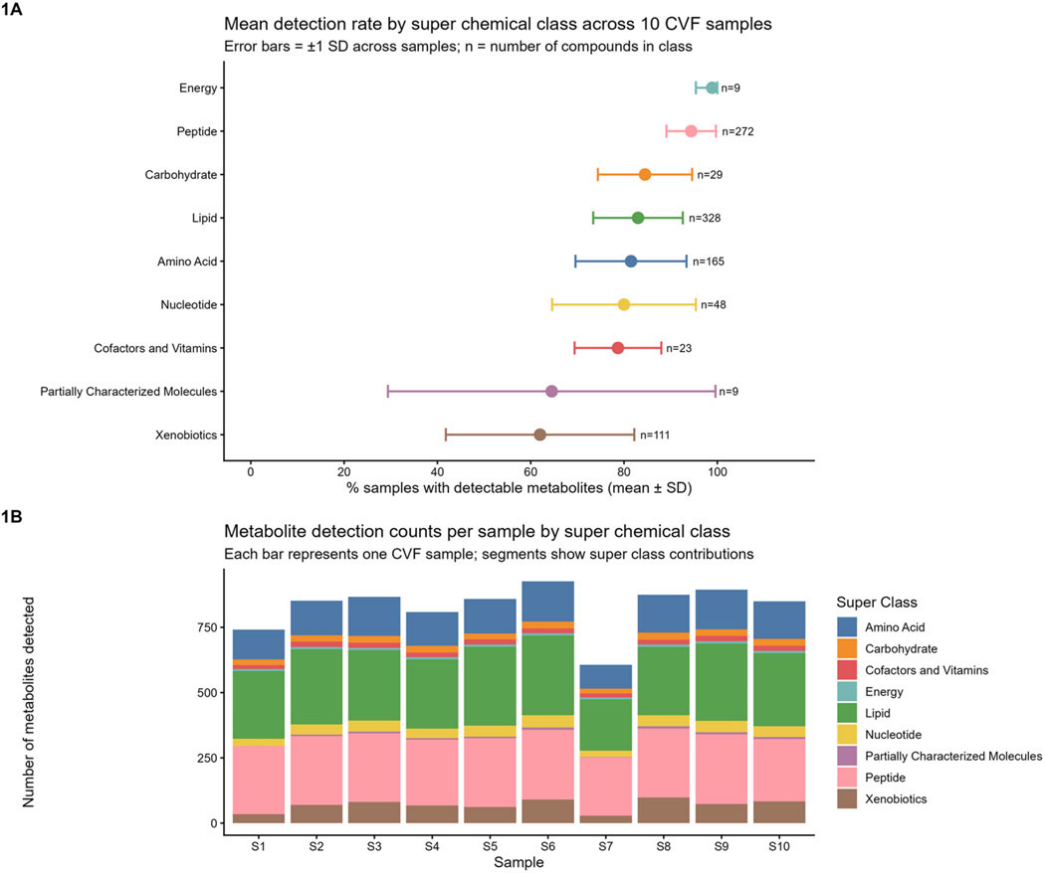
Metabolite detection rates and per-sample profiles across 10 CVF samples. *(1A)* mean detection rate (%) ± 1 standard deviation (SD) per super chemical class across the 10 samples. Energy metabolites (*n* = 9) and peptides (*n* = 272) showed the highest mean detection rates with the narrowest SDs (approaching 100%), while xenobiotics (*n* = 111) and partially characterized molecules (*n* = 9) showed the greatest inter-sample variability. Numbers indicate total compounds per super class. *(B)* Stacked bar chart showing total metabolite detection counts for each of the 10 CVF samples, with bar segments colored by super chemical class. Total detection counts ranged from 708 (S7) to 1035 (S6) metabolites per sample. Class proportions were broadly consistent across samples, with lipids and peptides dominating in all 10 participants. S1–S10 denote individual participant samples. CVF = cervicovaginal fluid.

Detection rates were consistently high across most super chemical classes. Energy metabolites showed the highest detection rate, with 99% of samples containing detectable levels of all 9 identified energy-related compounds. Peptides (*n* = 272) were detected in 94% of samples, followed by carbohydrates (*n* = 29, 84%), lipids (*n* = 328, 83%), amino acids (*n* = 165, 82%), nucleotides (*n* = 48, 80%), and cofactors and vitamins (*n* = 23, 79%). Even the less commonly detected super classes showed substantial presence, with partially characterized molecules detected in 64% of samples (*n* = 9) and xenobiotics in 62% of samples (*n* = 111). Lipids represented the largest super class with 328 distinct compounds detected, demonstrating the lipid-rich nature of CVF and the capacity of this approach to capture diverse metabolite profiles. Energy metabolites and Peptides showed the highest and most consistent detection rates across samples (mean detection rates of 99% and 94%, respectively, with narrow standard deviations), while Xenobiotics and Partially Characterized Molecules showed the greatest inter-sample variability (mean detection rate ± SD: 65% ± 23 and 64% ± 35%, respectively), consistent with expected inter-individual variation in exogenous exposures and incompletely characterized biochemicals (Figure 1A).

**Figure 2.**
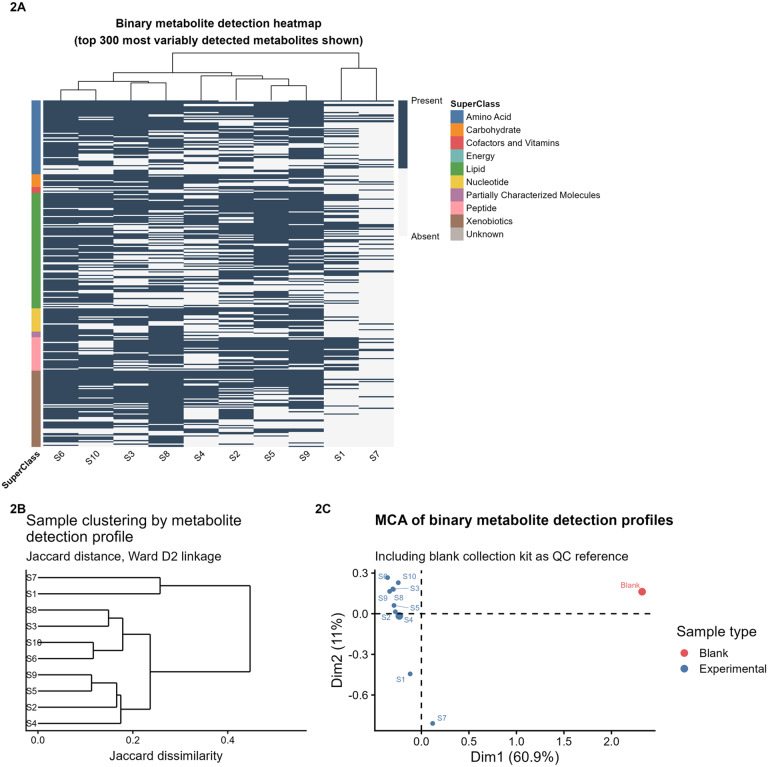
Binary metabolite detection profiles, sample clustering, and quality control validation in cervicovaginal fluid (CVF) from 10 ovarian cancer patients. *(2A)* binary detection heatmap displaying presence (dark slate) or absence (light gray) of the 300 most variably detected metabolites (those detected in 1–9 of 10 samples) across all samples. Rows are sorted by super chemical class (color bar, left) and columns are hierarchically clustered by binary Jaccard distance (Ward D2 linkage). Metabolite presence/absence was determined by UPLC-MS/MS using the Metabolon platform; compounds detected in the blank collection kit were excluded prior to analysis. *(B)* Hierarchical clustering dendrogram of the 10 CVF samples using Jaccard distance (Ward D2 linkage). Eight samples cluster tightly at low dissimilarity values (Jaccard ≤0.25), while S1 and S7, the two samples with the lowest total metabolite counts (846 and 708, respectively), form a distinct outgroup consistent with lower biological material on those swabs. Clustering topology was identical across Jaccard and Sørensen distance metrics (cophenetic *r* = 1.00; not shown). *(C)* Multiple correspondence analysis (MCA) of binary metabolite detection profiles including the blank collection kit as a quality control reference. The blank occupies a distinctly separate ordination space from all 10 study samples along dimension 1 (60.9% of variance), confirming that the 1,107 retained compounds represent donor-derived biological signal rather than kit background. S1–S10 denote individual participant samples. UPLC-MS = ultrahigh performance liquid chromatography-tandem mass spectroscopy.

Total metabolite detection counts ranged from 708 to 1035 per sample. Class proportions were broadly consistent across all 10 samples, with Lipids and Peptides dominating throughout (Figure 1B). Two samples with lower total counts showed proportionally similar class compositions to the remaining eight, suggesting reduced biological material on the swab rather than qualitative differences in metabolite profile, consistent with Metabolon’s range-finding documentation for swab-based specimens. The binary detection heatmap further illustrates the consistency of metabolite detection profiles across samples, with the majority of variably detected metabolites showing coherent patterns within super chemical classes (Figure 2A). Sample clustering based on binary detection profiles revealed that 8 of 10 samples grouped tightly at low dissimilarity values (Jaccard ≤0.25), while two samples with the lowest total metabolite counts (*n* = 846 and *n* = 708) formed a distinct outgroup (Figure 2B). The cophenetic correlation between Jaccard and Sørensen distance dendrograms was *r* = 1.00, confirming robustness of sample clustering to binary distance metric choice. MCA of binary detection profiles confirmed that the blank collection kit occupied a distinctly separate ordination space from all 10 study samples along Dimension 1 (60.9% of variance), validating that retained compounds represent donor-derived biological signal (Figure 2C).

**Figure 3.**
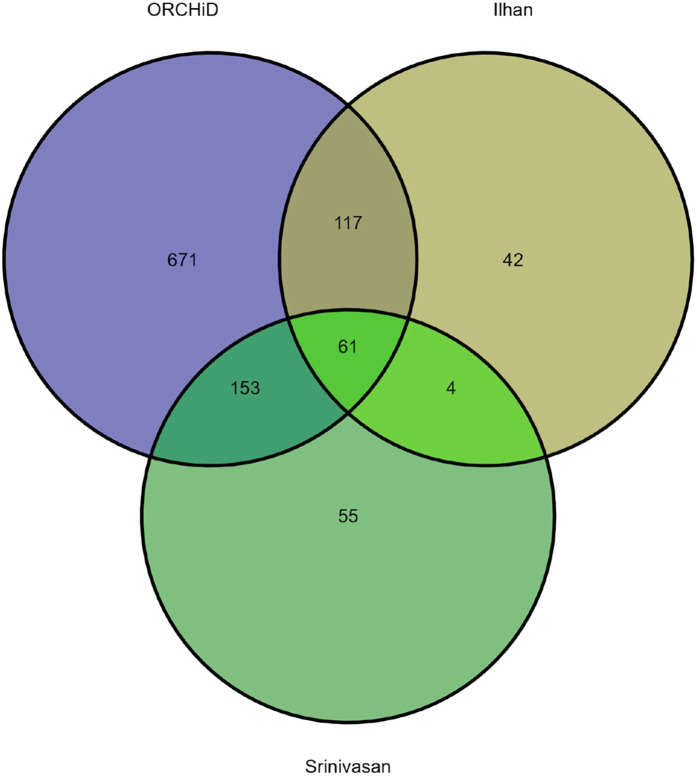
Overlap of detected metabolites between the ORCHiD pilot study and two published cervicovaginal fluid metabolomics studies. Venn diagram showing overlap of metabolites detected in the current study (ORCHiD, *n* = 10 patients with ovarian cancer) and two published CVF metabolomics studies: Srinivasan et al. 2015 (bacterial vaginosis cohort, *n* = 60) [10] and Ilhan et al. 2019 (cervical cancer cohort, *n* = 78) [11]. The 1107 chemical compounds detected in ORCHiD samples mapped to 1,002 unique RefMet identifiers following standardization using the Metabolomics Workbench RefMet database (accessed April 2026). Of these, 671 (66.9%) were uniquely detected in ORCHiD and not reported in prior publications. A core set of 61 metabolites (6.1%) were detected across all three studies despite differences in disease context, collection method, and analytical platform. An additional 153 (15.3%) and 117 (11.7%) metabolites overlapped specifically with Srinivasan et al. 2015 and Ilhan et al. 2019, respectively. ORCHiD = Ovarian Cancer Epidemiology, Healthcare Access and Disparities.

Of the 1,002 metabolites detected in our pilot study, 331 (33.0%) overlapped with prior publications (Figure 3), validating that samples collected in DNA/RNA preservation kits capture known CVF metabolites. Notably, 671 metabolites (66.9%) were uniquely detected in our study and have not been previously reported in CVF, demonstrating substantial potential for novel metabolite discovery. A core set of 61 metabolites (6.1%) was consistently detected across all three studies despite differences in disease context (ovarian cancer, bacterial vaginosis (BV), and cervical cancer), collection methods (self-collected vaginal swabs vs. clinician-collected lateral vaginal wall swabs vs. clinician-collected cervicovaginal lavage), and analytical platforms (LC/MS only vs combined GC/MS and LC/MS). As shown in Figure 3, an additional 153 metabolites (15.3%) overlapped specifically with Srinivasan et al. 2015 and 117 metabolites (11.7%) with Ilhan et al. 2019, further supporting the reproducibility and validity of untargeted metabolomics in our study design. Complete metabolite detection data with cross-study overlap annotations are available in Supplementary Table 1.

## Discussion

This study demonstrates the feasibility of conducting untargeted metabolomics on CVF samples originally collected for epidemiologic research using DNA/RNA preservation kits. We successfully detected 1107 distinct chemical compounds spanning 9 super chemical classes, with consistently high detection rates across most metabolite classes. Comparison with two published CVF metabolomics studies showed one-third of our detected metabolites overlapped with prior literature despite differences in collection methods and disease contexts. Notably, 61 core metabolites were detected across all three studies, demonstrating reproducibility even with different collection protocols. These findings suggest that self-collected samples and existing biospecimen protocols can enable cost-effective retrospective metabolomics studies, maximizing research value.

The 153 metabolites shared specifically between our study and Srinivasan et al. 2015, but not Ilhan et al. 2019, likely reflect a mix of core CVF metabolites present regardless of disease context and microbiome-influenced compounds. The Srinivasan cohort included women with and without BV, a condition characterized by overgrowth of anaerobic bacteria that produces characteristic metabolic byproducts including short-chain fatty acids, biogenic amines, and certain amino acid catabolites. Detection of these compounds in our study may reflect background vaginal microbiome variation among participants, independent of ovarian cancer status. Disentangling BV-associated from cancer-associated metabolite signatures would require concurrent microbiome profiling, which was not performed in this pilot, and represents an important direction for future work.

However, several limitations warrant consideration. This pilot study included only 10 samples from patients with ovarian cancer collected post-treatment, and the absence of non-ovarian cancer controls limits our ability to attribute uniquely detected metabolites to ovarian cancer specifically; they may instead reflect collection method differences, platform sensitivity, or post-treatment clinical context. Although self- and clinician-collected vaginal samples have shown concordance for microbiome composition [22,23], differences in insertion depth, technique, and timing relative to the menstrual cycle or sexual activity may still introduce variability [24,25]. Collection adequacy was not assessed at the individual sample level, and future studies should incorporate adequacy metrics to minimize this source of variability. Individual study samples were not run in technical replicate, consistent with the range-finding design; future quantitative studies should incorporate individual-sample replication. The DNA Genotek OMR-130 kit contains a proprietary stabilizing solution optimized for nucleic acid preservation rather than metabolomics, which may affect certain metabolite classes. Compounds most likely to be affected include volatile organic compounds and certain lipid subclasses susceptible to degradation during stabilization and storage. Conversely, amino acids, nucleotides, and most peptides are generally stable under preservation conditions, consistent with the high and consistent detection rates observed for these classes in our data. The robust detection of stable compound classes and strong overlap with prior CVF studies using different collection protocols suggest that preservation-related metabolite loss did not substantially compromise the core metabolite profile. Finally, this pilot sample was not fully demographically representative; future metabolomics analyses within ORCHiD should prioritize comprehensive demographic capture and diverse recruitment.

Despite these limitations, the robust detection of diverse metabolite classes and strong overlap with published CVF studies support the validity of this approach. Future investigations with larger sample sizes are needed to examine metabolite signatures associated with specific clinical characteristics, treatment responses, or disease outcomes in ovarian cancer. In addition, novel insights into cancer biology may be gleaned by examining metabolomic profiles of patients across diverse backgrounds and settings, insights that are uniquely enabled by embedding cost-effective biospecimen collection within population-based recruitment strategies. Critical appraisal by metabolomics experts remains essential when applying untargeted metabolomics to opportunistically collected samples to ensure data quality and scientific validity.

## Supporting information

10.1017/cts.2026.10766.sm001 Osazuwa-Peters et al. supplementary material Osazuwa-Peters et al. supplementary material

## Data Availability

Summary data are provided in Figures 1–3. Complete metabolite detection data for all 1,107 compounds from this pilot study, including cross-study overlap annotations with Srinivasan et al. 2015 and Ilhan et al. 2019, are available in Supplementary Table.

## Supplementary material

The supplementary material for this article can be found at https://doi.org/10.1017/cts.2026.10766.
